# Comparison of definitions of bronchopulmonary dysplasia to reflect the long-term outcomes of extremely preterm infants

**DOI:** 10.1038/s41598-022-22920-8

**Published:** 2022-10-27

**Authors:** Ga Won Jeon, Minkyung Oh, Juyoung Lee, Yong Hoon Jun, Yun Sil Chang

**Affiliations:** 1 Department of Pediatrics, Inha University Hospital, Inha University College of Medicine, Incheon, South Korea; 2 Department of Pharmacology, Inje University College of Medicine, Busan, South Korea; 3 Department of Pediatrics, Samsung Medical Center, Sungkyunkwan University School of Medicine, 81 Irwon-Ro, Gangnam-Gu, Seoul, 06351 South Korea; 4 Department of Health Sciences and Technology, Samsung Advanced Institute for Health Sciences & Technology (SAIHST), Samsung Medical Center, Seoul, Korea

**Keywords:** Preterm birth, Paediatric research, Neonatology

## Abstract

Survivors of neonatal bronchopulmonary dysplasia (BPD) have persistent respiratory, neurodevelopmental, and growth impairment over the first few years of life and later childhood, which represents an emerging burden for health systems. Therefore, there is an increasing need for a new definition and grading system of BPD that predicts long-term outcomes of high-risk infants who need timely and proper intervention to improve outcomes. We compared new definitions of BPD (National Institute of Child Health and Human Development [NICHD] 2018 and Neonatal Research Network [NRN] 2019) to the original NICHD 2001 definition at 3 years of age using a nationwide cohort of extreme preterm infants. New definitions and severity grading were clearly related to respiratory, neurodevelopmental, and growth impairments at 3 years of age and at 18–24 months corrected age (CA), whereas the original NICHD 2001 definition was not. Furthermore, the negative effect of BPD on growth was ameliorated at 3 years of age compared to 18–24 months CA. However, the negative effect of BPD in neonates on the respiratory system and neurodevelopment persisted at 3 years of age. These new definitions should be adopted to identify high-risk infants and improve long-term outcomes by exact diagnosis and BPD severity classification.

## Introduction

Recent studies have shown that preterm infants who require invasive ventilation at 36 weeks’ postmenstrual age (PMA) have an increased risk of mortality, neurodevelopmental impairment, and other comorbidities^1–3^. Extremely preterm infants (EPIs) whose gestational age is < 28 weeks are the high-risk group of bronchopulmonary dysplasia (BPD). BPD occur in up to 50% of infants born with a birth weight of < 1000 g^4^. Especially, severe BPD occurs in 42% of infants born with a birth weight of 501–750 g, and 25% of infants born with a birth weight of 751–1000 g^5^.

The wide spectrum of definitions used to classify BPD among neonatologists can cause variance in its incidence across centers. The definition of BPD should be practical, easy to apply, and predictive of long-term outcomes to identify high-risk infants with poor outcomes earlier. The diagnostic criteria of BPD have evolved since it was first described in the 1960s by Northway et al.^6–9^. The most widely used definition of BPD is the National Institute of Child Health and Human Development (NICHD) definition suggested in 2001^10^. However, this definition cannot predict long-term outcomes of EPIs and new respiratory management, such as high-flow nasal cannula^11^, is not addressed to BPD by this definition. There is a need to predict high-risk infants who require early intervention with a comprehensive and multidisciplinary approach to improve long-term outcomes. Survivors of neonatal BPD have persistent respiratory, neurodevelopmental, and growth impairment over the first few years of life, even in later childhood^12^, and represent an emerging burden and challenge for health systems. According to the requirement of accurate and timely definition with severity grading, new definition of BPD has emerged, and the two commonly used ones are the NICHD 2018 definition^13^ and NICHD Neonatal Research Network (NRN) 2019 definition by Jensen et al.^1^. There is no requirement for oxygen or respiratory support for the first 28 days of life, and BPD is assessed at 36 weeks’ PMA using these two definitions. BPD is graded using both oxygen concentration and respiratory support at 36 weeks’ PMA by the NICHD 2018 definition, which is graded using only respiratory support regardless of oxygen concentration at 36 weeks’ PMA by NRN 2019 definition.

We previously showed that recently suggested BPD definitions and grading of severity (NICHD 2016 and NRN 2019) better reflected morbidities at the corrected age (CA) of 18–24 months than the original NICHD 2001 definition^2^. However, since BPD survivors have persistent morbidities over the first few years of life, more long-term childhood morbidities need to be evaluated. Thus, we aimed to determine whether the newly suggested BPD definitions still better reflect 3 years’ long-term morbidities than the original NICHD 2001 definition, and whether morbidities related to the BPD severity were overcome over time from CA 18–24 months to 3 years of age in EPIs.

Therefore, in the present study we compared long-term respiratory, neurodevelopmental, and growth outcomes at 3 years of age according to three BPD definitions (NICHD 2001, NICHD 2018, and NRN 2019) with those at CA of 18–24 months in the nationwide large cohort of EPIs.

## Results

The prevalence of BPD in EPIs in Korea were 95% (2262/2374), 53% (1256/2363), and 53% (1256/2363), respectively, by definition A, B, and C.

### Characteristics of the patients

A total of 2380 EPIs who survived and registered to Korean Neonatal Network (KNN) were enrolled. The mean gestational age was 25.8 ± 1.2 weeks, and the mean birth weight was 882.0 ± 194.2 g. Overall, male neonates made up 53.5% of patients, and 6.2% of the patients were small for gestational age (SGA). Maternal gestational diabetes mellitus (GDM) and pregnancy induced hypertension (PIH) were observed in 6.9% and 8.9% of cases, respectively. Apgar scores at 1 and 5 min were 4 (3–5) and 7 (5–7), respectively. Follow-up rate at follow-up 1 (CA of 18–24 months) and 2 (3 years of age) were 69.4 and 43.1%, respectively (Table 1). The characteristics of EPIs who completed follow-up and those who were lost-to-follow-up are shown in Supplementary Table 1. EPIs who completed follow-up had lower birth weight, younger gestational age, higher rates of SGA and maternal chorioamnionitis, and higher level of maternal education than those who were lost-to-follow-up.

**Table 1:** Characteristics of the patients who were registered in the Korean Neonatal Network.

	Total N = 2380
Gestational age, weeks	25.8 ± 1.2
23, n (%)	114/2380 (4.8)
24, n (%)	289/2380 (12.1)
25, n (%)	478/2380 (20.1)
26, n (%)	663/2380 (27.9)
27, n (%)	836/2380 (35.1)
Birth weight, g	882.0 ± 194.2
< 500 g, n (%)	45/2380 (1.9)
500– < 750 g, n (%)	570/2380 (24.0)
750– < 1000 g, n (%)	1096/2380 (46.1)
1000– < 1500 g, n (%)	669/2380 (28.1)
Male, n (%)	1274/2380 (53.5)
Small for gestational age, n (%)	147/2380 (6.2)
Apgar score at 1 min, median (IQR)	4 (3–5)
Apgar score at 5 min, median (IQR)	7 (5–7)
Antenatal steroids therapy, n (%)	1985/2338 (84.9)
Maternal chorioamnionitis	1036/2043 (50.7)
Maternal GDM	165/2380 (6.9)
Maternal PIH	211/2380 (8.9)
Maternal level of education	
Elementary school	5/2380 (0.2)
Middle school	24/2380 (1.0)
High school	452/2380 (19.0)
University, or more	1363/2380 (57.3)
Unknown	536/2380 (22.5)
Follow-up at corrected age of 18–24 months, n (%)	1508/2174 (69.4)
Follow-up at three years of age, n (%)	938/2174 (43.1)

Values are expressed as mean ± standard deviation or number (%). IQR, interquartile range; GDM, gestational diabetes mellitus; PIH, pregnancy induced hypertension.

### Respiratory morbidities

Eleven infants died due to respiratory causes between 36 weeks’ PMA and follow-up 1, and no more infants died between follow-ups 1 and 2. A total of 39% and 10% of infants were readmitted due to respiratory cause between discharge from the neonatal intensive care unit (NICU) and follow-up 1, and between follow-ups 1 and 2, respectively. Further, 10%, and 6% of infants were readmitted ≥ 3 times due to respiratory causes between discharge from the NICU and follow-up 1, and between follow-ups 1 and 2. Respiratory morbidities at follow-up 1 and 2 were 11%, and 7%, respectively (Table 2).

**Table 2:** Respiratory morbidities at a corrected age of 18–24 months and at 3 years of age according to the severity of bronchopulmonary dysplasia per each definition.

		Corrected age of 18–24 months		Three years of age	
		Number (%)	AOR (95% CI)	Number (%)	AOR (95% CI)
Definition A	None	5/67 (7.5)		1/40 (2.5)	
	Grade 1	47/606 (7.8)	0.95 (0.36, 2.50)	12/353 (3.4)	1.31 (0.17, 10.49)
	Grade 2	28/290 (9.7)	1.31 (0.48, 3.60)	10/184 (5.4)	2.18 (0.27, 17.80)
	Grade 3	81/545 (14.9)	2.13 (0.81, 5.65)	41/361 (11.4)	4.48 (0.58, 34.65)
	Total	161/1508 (10.7)	P for trend < 0.001	64/938 (6.8)	P for trend < 0.001
Definition B	None	57/706 (8.1)		15/414 (3.6)	
	Grade 1	30/316 (9.5)	1.29 (0.80, 2.08)	12/206 (5.8)	1.62 (0.73, 3.58)
	Grade 2	27/227 (11.9)	1.60 (0.96, 2.66)	13/147 (8.8)	2.24 (0.99, 5.06)
	Grade 3	46/247 (18.6)	2.91 (1.86, 4.55)	23/164 (14.0)	4.22 (2.07, 8.61)
	Total	160/1496 (10.7)	P for trend < 0.001	63/931 (6.8)	P for trend < 0.001
Definition C	None	57/706 (8.1)		15/414 (3.6)	
	Grade 1	24/230 (10.4)	1.47 (0.87, 2.47)	9/146 (6.2)	1.82 (0.77, 4.28)
	Grade 2	51/423 (12.1)	1.66 (1.09, 2.54)	23/283 (8.1)	2.17 (1.08, 4.38)
	Grade 3	28/137 (20.4)	3.30 (1.94, 5.62)	16/88 (18.2)	5.90 (2.64, 13.17)
	Total	160/1496 (10.7)	P for trend < 0.001	63/931 (6.8)	P for trend < 0.001

*AOR* adjusted odds ratio; *CI*, confidence interval.

ORs were adjusted for antenatal steroids therapy, surfactant use, gestational age, small for gestational age, intraventricular hemorrhage (≥ grade 3), periventricular leukomalacia, necrotizing enterocolitis (≥ stage 2), retinopathy of prematurity (requiring surgery), and sepsis using multivariable logistic regression.

Respiratory morbidities were defined as (1) death due to respiratory causes from 36 weeks’ postmenstrual age prior to follow-up 1 or between follow-ups 1 and 2; (2) (2) more than two readmissions due to respiratory causes between discharge from the neonatal intensive care unit and follow-up 1 or between follow-ups 1 and 2; or (3) the requirement of oxygen, mechanical ventilation, or tracheostomy at follow-up 1 or 2. Follow-up 1, follow-up at a corrected age of 18–24 months; follow-up 2, follow-up at 3 years of age.

By definition A, there was no increased risk for respiratory morbidities in infants with BPD compared to infants without BPD both at follow-ups 1 and 2.

By definition B, respiratory morbidities increased from 8% among infants without BPD to 19% among those with grade 3 BPD at follow-up 1. Furthermore, respiratory morbidities were 4 and 14% among infants without BPD and those with grade 3 BPD, respectively, at follow-up 2. The risk of respiratory morbidities increased according to the severity of BPD at follow-ups 1 and 2 (P for trend < 0.001). Infants with grade 3 BPD had 2.91 times (95% confidence interval [CI] 1.86, 4.55) higher risk for respiratory morbidities compared to infants without BPD at follow-up 1. Furthermore, infants with grade 3 BPD had 4.22 times (95% CI 2.07, 8.61) higher risk for respiratory morbidities compared to infants without BPD at follow-up 2.

By definition C, respiratory morbidities increased from 8% among infants without BPD to 20% among those with grade 3 BPD at follow-up 1. Respiratory morbidities were 4 and 18% among infants without BPD and those with grade 3 BPD, respectively, at follow-up 2. The risk of respiratory morbidities increased according to the severity of BPD at follow-ups 1 and 2 (P for trend < 0.001). Infants with grade 2 and 3 BPD had 1.66 times (95% CI 1.09, 2.54) and 3.30 times (95% CI 1.94, 5.62) higher risk for respiratory morbidities compared to infants without BPD at follow-up 1, respectively. And, infants with grade 2 and 3 BPD had 2.17 times (95% CI 1.08, 4.38) and 5.90 times (95% CI 2.64, 13.17) higher risk for respiratory morbidities compared to infants without BPD at follow-up 2, respectively (Table 2).

### Neurodevelopmental impairments

A total of 22, 24, and 6% of infants had mental developmental delay, motor developmental delay, and social developmental delay at follow-up 1. Overall, 23, 27, and 13% of infants had mental developmental delay, motor developmental delay, and social developmental delay, respectively, at follow-up 2. Neurodevelopmental impairments at follow-up 1 and 2 were 33, and 36%, respectively (Table 3).

**Table 3:** Neurodevelopmental impairments at a corrected age of 18–24 months and at 3 years of age according to the severity of bronchopulmonary dysplasia per each definition.

		Corrected age of 18–24 months		Three years of age	
		Number (%)	AOR (95% CI)	Number (%)	AOR (95% CI)
Definition A	None	13/60 (21.7)		8/39 (20.5)	
	Grade 1	118/520 (22.7)	0.86 (0.44, 1.70)	75/284 (26.4)	1.34 (0.56, 3.21)
	Grade 2	67/238 (28.2)	1.01 (0.50, 2.07)	40/137 (29.2)	1.32 (0.52, 3.32)
	Grade 3	223/469 (47.6)	1.87 (0.94, 3.71)	152/300 (50.7)	2.69 (1.11, 6.52)
	Total	421/1287 (32.7)	P for trend < 0.001	275/760 (36.2)	P for trend < 0.001
Definition B	None	145/610 (23.8)		91/341 (26.7)	
	Grade 1	81/264 (30.7)	1.06 (0.75, 1.51)	56/163 (34.4)	1.04 (0.66, 1.63)
	Grade 2	92/196 (46.9)	1.99 (1.37, 2.88)	57/121 (47.1)	1.70 (1.04, 2.78)
	Grade 3	99/209 (47.4)	1.96 (1.36, 2.82)	69/130 (53.1)	2.24 (1.39, 3.60)
	Total	417/1279 (32.6)	P for trend < 0.001	273/755 (36.2)	P for trend < 0.001
Definition C	None	145/610 (23.8)		91/341 (26.7)	
	Grade 1	49/188 (26.1)	0.91 (0.61, 1.36)	31/108 (28.7)	0.85 (0.50, 1.43)
	Grade 2	161/372 (43.3)	1.73 (1.27, 2.35)	110/238 (46.2)	1.76 (1.18, 2.62)
	Grade 3	62/109 (56.9)	2.74 (1.72, 4.34)	41/68 (60.3)	2.38 (1.30, 4.35)
	Total	417/1279 (32.6)	P for trend < 0.001	273/755 (36.2)	P for trend < 0.001

*AOR* adjusted odds ratio; *CI* confidence interval.

ORs were adjusted for gestational age, small for gestational age, intraventricular hemorrhage (≥ grade 3), periventricular leukomalacia, necrotizing enterocolitis (≥ stage 2), retinopathy of prematurity (requiring surgery), and sepsis using multivariable logistic regression.

Neurodevelopmental impairment was defined as mental developmental delay, motor developmental delay, or social developmental delay.

By definition A, the risk for neurodevelopmental impairments in infants with BPD did not increase compared to the risk in infants without BPD at follow-up 1. Infants with grade 3 BPD had 2.69 times (95% CI 1.11, 6.52) higher risk for neurodevelopmental impairments compared to infants without BPD at follow-up 2.

By definition B, neurodevelopmental impairments increased from 24% among infants without BPD to 47% among those with grade 3 BPD at follow-up 1, while neurodevelopmental impairments were 27%, and 53% among infants without BPD and those with grade 3 BPD, respectively, at follow-up 2. Infants with grade 2 and 3 BPD had 1.99 times (95% CI 1.37, 2.88) and 1.96 times (95% CI 1.36, 2.82) higher risk for neurodevelopmental impairments compared to infants without BPD at follow-up 1. Furthermore, infants with grade 2 and 3 BPD had 1.70 times (95% CI 1.04, 2.78) and 2.24 times (95% CI 1.39, 3.60) higher risk for neurodevelopmental impairments compared to infants without BPD at follow-up 2, respectively.

By definition C, neurodevelopmental impairments increased from 24% among infants without BPD to 57% among those with grade 3 BPD at follow-up 1, while neurodevelopmental impairments were 27% and 60% among infants without BPD and those with grade 3 BPD, respectively, at follow-up 2. The risk of neurodevelopmental impairments increased according to the severity of BPD at follow-ups 1 and 2 (P for trend < 0.001). Infants with grade 2 and 3 BPD had 1.73 times (95% CI 1.27, 2.35) and 2.74 times (95% CI 1.72, 4.34) higher risk for neurodevelopmental impairments, respectively, compared to infants without BPD at follow-up 1. Infants with grade 2 and 3 BPD had 1.76 times (95% CI 1.18, 2.62) and 2.38 times (95% CI 1.30, 4.35) higher risk for neurodevelopmental impairments compared to infants without BPD at follow-up 2, respectively (Table 3).

### Growth restrictions

Growth restrictions at follow-up 1 and 2 were 38, and 10%, respectively.

By definition A, infants with grade 3 BPD had 2.70 times (95% CI 1.43, 5.11) higher risk for growth restrictions than infants without BPD at follow-up 1. However, there was not increased risk for growth restrictions in infants with BPD compared to infants without BPD at follow-up 2 (Table 4).

**Table 4:** Growth restrictions at a corrected age of 18–24 months and at 3 years of age according to the severity of bronchopulmonary dysplasia per each definition.

		Corrected age of 18–24 months		Three years of age	
		Number (%)	AOR (95% CI)	Number (%)	AOR (95% CI)
Definition A	None	15/66 (22.7)		1/39 (2.6)	
	Grade 1	170/592 (28.7)	1.26 (0.68, 2.36)	16/339 (4.7)	1.39 (0.18, 10.94)
	Grade 2	95/283 (33.6)	1.47 (0.77, 2.83)	10/174 (5.8)	1.65 (0.20, 13.54)
	Grade 3	282/528 (53.4)	2.70 (1.43, 5.11)	62/339 (18.3)	4.73 (0.62, 36.26)
	Total	562/1469 (38.3)	P for trend < 0.001	89/891 (10.0)	P for trend < 0.001
Definition B	None	195/690 (28.3)		18/398 (4.5)	
	Grade 1	127/311 (40.8)	1.47 (1.09, 1.97)	17/197 (8.6)	1.70 (0.84, 3.45)
	Grade 2	108/222 (48.7)	1.68 (1.20, 2.35)	25/143 (17.5)	3.13 (1.60, 6.13)
	Grade 3	126/234 (53.9)	2.21 (1.59, 3.07)	28/146 (19.2)	3.85 (1.99, 7.45)
	Total	556/1457 (38.2)	P for trend < 0.001	88/884 (10.0)	P for trend < 0.001
Definition C	None	195/690 (28.3)		18/398 (4.5)	
	Grade 1	83/226 (36.7)	1.31 (0.94, 1.83)	9/139 (6.5)	1.42 (0.61, 3.30)
	Grade 2	201/415 (48.4)	1.77 (1.34, 2.33)	38/270 (14.1)	2.59 (1.41, 4.78)
	Grade 3	77/126 (61.1)	2.82 (1.85, 4.30)	23/77 (29.9)	6.47 (3.12, 13.42)
	Total	556/1457 (38.2)	P for trend < 0.001	88/884 (10.0)	P for trend < 0.001

*AOR* adjusted odds ratio; *CI* confidence interval.

ORs were adjusted for gestational age, small for gestational age, intraventricular hemorrhage (≥ grade 3), periventricular leukomalacia, necrotizing enterocolitis (≥ stage 2), retinopathy of prematurity (requiring surgery), and sepsis using multivariable logistic regression.

Growth restriction was defined as z-scores <  − 1.28 (equivalent to < 10th percentile) of weight, height, or head circumference by the 2006 World Health Organization Child Growth Standards, meaning failure of catch-up growth.

By definition B, growth restrictions increased from 28% among infants without BPD to 54% among those with grade 3 BPD at follow-up 1, which were decreased to 5 and 19% among infants without BPD and among those with grade 3 BPD, respectively, at follow-up 2. The risk of growth restrictions increased according to the severity of BPD at follow-ups 1 and 2 (P for trend < 0.001). Infants with grade 1, 2 and 3 BPD had 1.47 (95% CI 1.09, 1.97), 1.68 (95% CI 1.20, 2.35), and 2.21 (95% CI 1.59, 3.07) times higher risk for growth restrictions compared to infants without BPD at follow-up 1, respectively. Infants with grade 2 and 3 BPD had 3.13 times (95% CI 1.60, 6.13) and 3.85 times (95% CI 1.99, 7.45) higher risk for growth restrictions compared to infants without BPD at follow-up 2, respectively.

By definition C, growth restrictions increased from 28% among infants without BPD to 61% among those with grade 3 BPD at follow-up 1, which were decreased to 5 and 30% among infants without BPD and among those with grade 3 BPD, respectively, at follow-up 2. The risk of growth restrictions increased according to the severity of BPD at follow-ups 1 and 2 (P for trend < 0.001). Infants with grade 2 and 3 BPD had 1.77 times (95% CI 1.34, 2.33) and 2.82 times (95% CI 1.85, 4.30) higher risk for growth restrictions, respectively, compared to infants without BPD at follow-up 1. Infants with grade 2 and 3 BPD had 2.59 times (95% CI 1.41, 4.78) and 6.47 times (95% CI 3.12, 13.42) higher risk for growth restrictions, respectively, compared to infants without BPD at follow-up 2, respectively.

### Association of the severity of BPD and the risk of long-term outcomes over time

To account for repeated measures and missing data, we reanalyzed the data with generalized estimating equation (GEE) models. Infants with BPD did not have higher risk of respiratory morbidities in all BPD severities by definition A (Table 5). By definition B and C, infants with grade 3 BPD had 2.61 times (95% CI 1.26, 5.41) and 3.10 times (95% CI 1.23, 7.77) higher risk for respiratory morbidities, respectively, compared to infants without BPD. By definition A, infants with grade 3 BPD had 2.37 times (95% CI 1.08, 5.16) higher risk for neurodevelopmental impairments compared to infants without BPD. By definitions B and C, infants with grade 2 and 3 BPD had higher risk for neurodevelopmental impairments compared to infants without BPD (2.23 times [95% CI 1.48, 3.37] and 2.37 times [95% CI 1.51, 3.71] in grade 2 and 3 BPD, respectively, by definition B; 2.00 times [95% CI 1.41, 2.84] and 3.10 times [95% CI 1.78, 5.41] in grade 2 and 3 BPD, respectively, by definition C). By definition A, infants with grade 3 BPD had 2.54 times (95% CI 1.22, 5.29) higher risk for growth restrictions compared to infants without BPD. By definitions B and C, infants with grade 2 and 3 BPD had higher risk for growth restrictions compared to infants without BPD (1.96 times [95% CI 1.32, 2.89] and 2.12 times [95% CI 1.44, 3.12] in grade 2 and 3 BPD, respectively, by definition B; 1.93 times [95% CI 1.37, 2.71] and 2.60 times [95% CI 1.58, 4.28] in grade 2 and 3 BPD, respectively, by definition C). When analyzing the interaction between BPD severity and time between follow-up 1 and 2, the risk of growth restriction decreased from follow-up 1 to follow-up 2 by all definitions (adjusted odds ratio [AOR] 0.08 [95% CI 0.01, 0.63], AOR 0.10 [95% CI 0.06, 0.17], and AOR 0.10 [95% CI 0.06, 0.18] in definition A, B, and C, respectively). The risk of respiratory morbidities or neurodevelopmental impairments were not decreased from follow-up 1 to follow-up 2 (Table 5).

**Table 5:** Association of the severity of bronchopulmonary dysplasia and the risk of long-term outcomes over time per each definition.

Main effect: the severity of bronchopulmonary dysplasia		Respiratory morbidities		Neurodevelopmental impairments		Growth restrictions	
		AOR (95% CI)	*P* value	AOR (95% CI)	P value	AOR (95% CI)	*P* value
Definition A	Grade 1	1.28 (0.26, 6.34)	0.760	0.94 (0.45, 1.98)	0.879	1.19 (0.56, 2.52)	0.652
	Grade 2	2.03 (0.42, 9.83)	0.381	1.03 (0.49, 2.15)	0.939	1.25 (0.59, 2.64)	0.565
	Grade 3	2.71 (0.58, 12.74)	0.206	2.37 (1.08, 5.16)	0.031	2.54 (1.22, 5.29)	0.013
	Time	0.49 (0.04, 6.34)	0.583	1.02 (0.36, 2.89)	0.972	0.08 (0.01, 0.63)	0.016
Definition B	Grade 1	1.45 (0.75, 2.78)	0.266	1.10 (0.72, 1.66)	0.661	1.37 (0.96, 1.94)	0.079
	Grade 2	1.58 (0.79, 3.17)	0.193	2.23 (1.48, 3.37)	< 0.001	1.96 (1.32, 2.89)	0.001
	Grade 3	2.61 (1.26, 5.41)	0.010	2.37 (1.51, 3.71)	< 0.001	2.12 (1.44, 3.12)	< 0.001
	Time	0.52 (0.26, 1.03)	0.059	1.36 (0.95, 1.95)	0.098	0.10 (0.06, 0.17)	< 0.001
Definition C	Grade 1	1.62 (0.81, 3.25)	0.176	0.88 (0.53, 1.46)	0.621	1.17 (0.79, 1.76)	0.434
	Grade 2	1.63 (0.89, 2.97)	0.112	2.00 (1.41, 2.84)	< 0.001	1.93 (1.37, 2.71)	< 0.001
	Grade 3	3.10 (1.23, 7.77)	0.016	3.10 (1.78, 5.41)	< 0.001	2.60 (1.58, 4.28)	< 0.001
	Time	0.52 (0.26, 1.03)	0.060	1.36 (0.95, 1.95)	0.096	0.10 (0.06, 0.18)	< 0.001

*AOR* adjusted odds ratio; *CI* confidence interval.

ORs were adjusted for gestational age, small for gestational age, intraventricular hemorrhage (≥ grade 3), periventricular leukomalacia, necrotizing enterocolitis (≥ stage 2), retinopathy of prematurity (requiring surgery), and sepsis.

A generalized estimating equation (GEE) method was applied to find the association between the severity of bronchopulmonary dysplasia and the risk of long-term outcomes over time per each definition.

## Discussion

In this study, infants with BPD by definition A (NICHD 2001 definition) did not show higher risk for long-term respiratory, neurodevelopmental, and growth outcomes. However, infants with BPD by definition B (modified NICHD 2018 definition) and C (modified NRN 2019 definition) showed increased risk for poor long-term respiratory, neurodevelopmental, and growth outcomes at 3 years of age as well as at CA of 18–24 months according to the severity of BPD.

In Korea, 65.7% (2153/3277) of EPIs with a gestational age of 23 to 27 weeks born between 2014 and 2017 died before 36 weeks’ PMA or developed BPD (by definition B/C) in the present study. In 2018, reports from the United States showed that 51% of infants born at a gestational age of 22–29 weeks died before 36 weeks’ PMA or developed BPD^14^. This means clearly that BPD is a big burden for the society and health systems.

To improve long-term outcomes of EPIs with BPD who have high risks of respiratory, neurodevelopmental, and growth impairments, the definition of BPD has evolved to predict high-risk infants who require early interventions.

The most widely used definition is the one proposed by the NICHD workshop in 2001^10^. This definition was proposed more than 20 years ago when survival rates of EPIs were extremely low. Nowadays, together with the markedly improved survival, the surviving EPIs require oxygen for a long duration, sometimes more than 28 days. These EPIs were diagnosed at least with “mild BPD” according to the NICHD 2001 definition. However, long-term outcomes of infants with “mild BPD,” according to this definition, were comparable to those of infants without BPD. Therefore, we did not need to classify these infants as having BPD. Furthermore, infants with a high-flow nasal cannula or recently developed respiratory support cannot be classified according to the NICHD 2001 definition. Thus, this definition is no longer useful for EPIs. Furthermore, the new definition proposed in the NICHD workshop in 2016^13^ had better predictive ability at CA of 20–24 months^15^, that also had higher specificity and better positive predictive value at CA of 18–24 months^16^ than the NICHD 2001 definition. The NICHD 2018 definition diagnose BPD on respiratory support and oxygen concentration at 36 weeks’ PMA without requiring oxygen for the first 28 days of life. However, this definition is not easy to use and somewhat complicated (Table 6). The NICHD NRN proposed definition in 2019^1^ only depends on respiratory support at 36 weeks’ PMA, which depends on neither oxygen concentration at 36 weeks’ PMA nor oxygen requirement for the first 28 days of life. This definition is easy to apply, and can predict late death or long-term respiratory morbidities (predictive accuracy 0.785) as well as late death or long-term neurodevelopmental impairments (predictive accuracy 0.747) at CA of 18–26 months^1^. Grade 3 BPD according to the NRN 2019 definition (which is dependent on invasive ventilator [IV]) had stronger association with mortality, tracheostomy, or hospital duration in preterm infants with gestational age < 32 weeks compared to the NICHD 2018 definition^17^. Furthermore, exposure to mechanical ventilation is associated with the development of grade 2/3 BPD (by NICHD 2001 definition) in preterm infants with gestational age < 30 weeks^18^. This implies that mechanical ventilation is a more important risk factor related to BPD than oxygen concentration. Type 2 severe BPD (corresponding to grade 3 BPD according to NRN 2019 definition) requiring IV at 36 weeks’ PMA was associated with increased risk of mortality, tracheostomy, or gastrostomy compared to type 1 severe BPD (corresponding to grade 1 or 2 BPD according to NRN 2019 definition)^19,20^. This result also implies that dependency on IV at 36 weeks’ PMA (grade 3 BPD by NRN 2019 definition) is the most important prognostic factor for mortality and outcomes.

**Table 6:** Definitions of bronchopulmonary dysplasia.

	Definition A and NICHD 2001 definition	
Grade 1	(O_2_, FiO_2_ > 21% for ≥ 28 days and); breathes room air	
Grade 2	O_2_, FiO_2_ 0.22– < 0.3	
Grade 3	O_2_, FiO_2_ ≥ 0.3 Positive pressure ventilator	
	Definition B	NICHD 2018 definition
Grade 1	O_2_ < 2L/min, FiO_2_ > 0.21 NIV (O_2_ ≥ 2L/min), FiO_2_ 0.21	O_2_ < 1L/min, FiO_2_ > 0.21 (– ≤ 0.7) O_2_ 1– < 3L/min, FiO_2_ 0.22– < 0.3 NIV (O_2_ ≥ 3L/min), FiO_2_ 0.21
Grade 2	NIV (O_2_ ≥ 2L/min), FiO_2_ 0.22– < 0.3 IV, FiO_2_ 0.21	O_2_ < 1L/min, FiO_2_ > 0.7 O_2_ 1– < 3L/min, FiO_2_ ≥ 0.3 NIV (O_2_ ≥ 3L/min), FiO_2_ 0.22– < 0.3 IV, FiO_2_ 0.21
Grade 3	NIV (O_2_ ≥ 2L/min), FiO_2_ ≥ 0.3 IV, FiO_2_ > 0.21	NIV (O_2_ ≥ 3L/min), FiO_2_ ≥ 0.3 IV, FiO_2_ > 0.21
	Definition C	NRN 2019 definition
Grade 1	O_2_ < 2L/min	O_2_ ≤ 2L/min
Grade 2	NIV (O_2_ ≥ 2L/min)	NIV (O_2_ > 2L/min)
Grade 3	IV	IV

*NICHD* National Institute of Child Health and Human Development; *NIV* non-invasive ventilator; *IV* invasive ventilator; *NRN* Neonatal Research Network.

Adult survivors of neonatal BPD show greater deficits in executive functioning related to problem solving, awareness of behavior, and organization of environment compared to preterm infants without BPD or term infants^21^. Though neurodevelopmental, respiratory, or growth impairments in BPD survivors extend into adulthood in those with great deficits, there are few studies that compare long-term morbidities at certain time points, such as at a CA of 18–24 months and 3 years of age like our present study.

Long-term motor developmental impairment both at 18–24 months of CA and at 3 years was found in EPIs with grade 3 BPD according to definitions B and C in the present study. Further, BPD was strongly associated with the increased risk of developmental delay at a CA of 24 months^22^. Also, 31.3% of preterm infants with BPD born at gestational age < 29 weeks had neurodevelopmental impairment at CA 2 years^23^. Together with long-term motor developmental impairment, severe BPD increased the risk of early motor developmental delay at CA of 3 and 6 months^24^. Hence, early precise diagnosis of BPD and identification of high-risk infants to facilitate earlier intervention is crucial to improve long-term outcomes. Neurodevelopmental impairments became more severe according to the progressive severity of BPD, which were aggravated at a CA of 5 years compared to a CA of 2 years by Katz et al.^25^. However, in the present study, the risk of neurodevelopmental impairments was not increased according to time, namely from a CA of 18–24 months to 3 years of age.

Respiratory morbidities were not improved at 3 years of age compared to a CA of 18–24 months in the present study. A total of 39% of EPIs with BPD readmitted because of pulmonary complications of BPD in the present study before reaching a CA of 18–24 months. Similarly, 49% of preterm infants with BPD were readmitted due to respiratory cause for the first year of life^26^. Furthermore, pulmonary dysfunction, exercise intolerance, or wheezing can persist in survivors of neonatal BPD during early childhood even in young adulthood due to the injury on respiratory system occurred during the neonatal period, though BPD tends to improve with lung development after birth^26,27^. This is why the risk of respiratory morbidities was not decreased at 3 years of age compared to at a CA of 18–24 months in the present study.

Growth restrictions were improved at 3 years of age compared to at a CA of 18–24 months in the present study. Growth restriction at a CA of 18–24 months in grade 3 BPD by definition B and C were 54 and 61%, which was decreased to 19 and 30% at 3 years of age, respectively. This might be explained by the aggressive nutritional strategies for infants with BPD^28^. EPIs with BPD usually have postnatal growth restriction, because of high metabolic demands with poor nutritional reserves. However, Dassios et al. reported that through the use of aggressive nutritional strategies, short-term outcomes of weight and head circumference growth in infants with BPD at 36 weeks’ PMA had overcome those of infants without BPD^28^.

This study has some limitations. First, because of the multi-center nature of this registry, each hospital uses different modalities to evaluate neurodevelopment, so inter-hospital variability or inter-test variability was inevitable. Furthermore, we developed the composite data to minimize these variability and selection bias. We developed the composite scores on motor, mental, and social domain using each item in Bayley Scales of Infant Development (BSID) II, BSID III, Korean Developmental Screening Test for Infants and Children (K-DST)^29^, or Korean Ages & Stages Questionnaires (K-ASQ)^30^ described in the section of method^2^. Second, modifications of the NICHD 2018 definition and the NRN 2019 definition to definitions B and C were inevitable, because the KNN pre-set registry data were used. The comparison between definition B, C, NICHD 2018, and NRN 2019 definition is presented in Table 6. Third, in the present study, the number of emergency room visits due to respiratory causes was not included in the respiratory morbidities, and an abnormal pulmonary function test was also not included because the KNN data did not comprise these types of data.

However, a strength of this study is the exact comparison of long-term respiratory, neurodevelopmental, and growth outcomes in EPIs with BPD in a nationwide prospective cohort at certain time points (at a CA of 18–24 months and at 3 years of age). There are few studies comparing long-term morbidities of such infants at certain time points. Our previous study^2^ concluded that the recently suggested BPD definitions better reflected long-term morbidities at a CA of 18–24 months than the original NICHD 2001 definition. The present study also confirmed this result at 3 years of age, in addition to a CA of 18–24 months. BPD survivors have persistent long-term morbidities during the first few years of life; therefore, there is a need to evaluate more long-term morbidities. Further, we aimed to determine whether long-term morbidities were overcome over time from CA of 18–24 months to 3 years of age. The rates of respiratory morbidities and growth restrictions at a CA of 18–24 months were 11 and 38%, respectively, which decreased to 7 and 10%, respectively, at 3 years of age, although without statistical comparison. In our previous study^2^, EPIs were enrolled between January 1, 2013 and December 31, 2015, while the present study enrolled more EPIs born between January 1, 2014 and December 31, 2017. Overlapping of some EPIs was unavoidable. Similar to a previous study with KNN data^31^, EPIs with lower birth weight, younger gestational age, SGA, and maternal chorioamnionitis were followed-up in the present study. It appears that there was at least no bias to exclude more severe EPIs in the present study performed with those who completed follow-up.

In conclusion, respiratory, neurodevelopmental, and growth impairments at 3 years of age as well as at a CA of 18–24 months were clearly associated with the increased severity of BPD according to the new definitions (definition B and C) not by the original NICHD 2001 definition. This negative effect of BPD on growth tends to be ameliorated with the growth of the infants. However, the negative effect of BPD occurred in neonatal period on respiratory system and neurodevelopment persisted at 3 years of age. So, these new definitions should be adopted to identify high-risk infants who require timely and proper intervention to improve long-term outcomes by exact diagnosis and classification of the severity of BPD.

## Methods

This prospective, nationwide, large cohort study included preterm infants in the 70 participating centers, covering > 80% of very low birth weight infants (VLBWIs) in Korea since KNN was launched in 2013^32^. A total of 3277 EPIs born at a gestational age of 23–27 weeks between January 1, 2014 and December 31, 2017 were registered to KNN, and 897 of them who died before being diagnosed with BPD at 36 weeks PMA or due to congenital anomalies were excluded. The 2380 EPIs who survived were enrolled, and among them, 1508 were followed up at a CA of 18–24 months (follow-up 1), and 938 were followed up at 3 years of age (follow-up 2). The baseline characteristics of the EPIs registered in the KNN were analyzed. Furthermore, the characteristics of EPIs who completed follow-up were compared with those of EPIs who were lost-to-follow-up. We evaluated long-term outcomes, namely, respiratory morbidities, neurodevelopmental impairment, and growth restriction both at follow-up 1 and 2.

BPD was assessed at 36 weeks’ PMA, which was classified to no, grade 1, 2, and 3 BPD according to severity by each definition (Table 6). Definition A is the commonly used NICHD 2001 definition^10^ which KNN uses. Grade 1 breathes room air, grade 2 needs oxygen with FiO_2_ 0.22– < 0.3, and grade 3 needs oxygen with FiO_2_ ≥ 0.3 or positive pressure ventilation with the precondition of oxygen at least during the first 28 days of life. We use the KNN pre-set registry data; therefore, modifications of the NICHD 2018 and NRN 2019 definitions are inevitable. Definition B is the modified NICHD 2018 definition^13^. Grade 1 breathes oxygen using a nasal cannula at < 2 L/min with FiO_2_ > 0.21, or noninvasive ventilator ([NIV], including a nasal cannula oxygen at ≥ 2 L/min) with FiO_2_ 0.21. Grade 2 breathes with NIV (including a nasal cannula oxygen ≥ 2 L/min) with FiO_2_ 0.22– < 0.3 or IV with FiO_2_ 0.21. Grade 3 BPD breathe with NIV (including a nasal cannula oxygen ≥ 2 L/min) with FiO_2_ ≥ 0.3, or IV with FiO_2_ > 0.21. NIV in the KNN registry (definition B) includes nasal cannula oxygen ≥ 2 L/min, whereas NIV in the NICHD 2018 definition includes nasal cannula oxygen ≥ 3 L/min. FiO2 is classified as 0.21, 0.22–0.29, and ≥ 0.3 in the KNN registry; in contrast, it is classified as 0.21, 0.22–0.69, and ≥ 0.7 with nasal cannula oxygen < 1 L/min and 0.21, 0.22–0.29, and ≥ 0.3 with nasal cannula oxygen ≥ 1 L/min or ventilator in the NICHD 2018 definition. The flow rate of the nasal cannula oxygen is classified as < 2 L/min and ≥ 2 L/min in the KNN registry, and as < 1 L/min, 1– < 3 L/min, and ≥ 3 L/min in the NICHD 2018 definition (Table 6). Definition C is the modified NRN 2019 definition^1^. Grade 1 BPD breathes oxygen using a nasal cannula at < 2 L/min. Grade 2 BPD breathe with NIV (including nasal cannula oxygen at ≥ 2 L/min). Grade 3 BPD needs IV. The only difference between definitions C and NRN 2019 is that NIV includes nasal cannula oxygen ≥ 2 L/min in the KNN registry, whereas, it includes nasal cannula oxygen > 2 L/min in the NRN 2019 definition (Table 6).

Respiratory morbidities were defined as (1) death due to respiratory causes from 36 weeks’ PMA prior to follow-up 1 or between follow-ups 1 and 2; (2) more than two readmissions due to respiratory causes between discharge from the NICU and follow-up 1 or between follow-ups 1 and 2; or (3) the requirement of oxygen, mechanical ventilation, or tracheostomy at follow-up 1 or 2.

Neurodevelopmental impairment was defined as mental developmental delay, motor developmental delay, or social developmental delay. Mental developmental delay was defined as (1) a mental developmental index (MDI) < 70 on the BSID II; (2) cognitive or language < 70 on the BSID III; (3) cognition, language, or self-help score that is less than the cut-off value on the K-DST; or (4) communication or problem-solving score that is less than the cut-off value on the K-ASQ. Motor developmental delay was defined as (1) a psychomotor developmental index (PDI) < 70 on the BSID II; (2) motor < 70 on the BSID III; (3) gross motor or fine motor score that is less than the cut-off value on K-DST or K-ASQ; or (4) cerebral palsy defined as Gross Motor Functional Classification System ≥ 2^33^. Social developmental delay was defined as (1) a sociality less than the cut-off value on the K-DST; or (2) a personal-social score less than the cut-off value on the K-ASQ.

Growth restriction was defined as z-scores <  − 1.28 (equivalent to < 10th percentile) of weight, height, or head circumference according to the CA by the 2006 World Health Organization (WHO) Child Growth Standards, meaning failure of catch-up growth^34^.

### Statistical analysis

Continuous variables were compared using the *t*-test or Mann–Whitney U test, and categorical variables were compared using the chi-square test or Fisher’s exact test, as appropriate. Continuous variables are presented as mean ± standard deviation (SD) and categorical variables are presented as number (percentage). Apgar scores are presented as median (interquartile range [IQR]).

ORs compared to no BPD according to various BPD severity per each definition using multivariable logistic regression were analyzed. ORs for neurodevelopmental impairment and growth restriction were adjusted for gestational age, SGA, intraventricular hemorrhage (≥ grade 3), periventricular leukomalacia, necrotizing enterocolitis (≥ stage 2), retinopathy of prematurity (requiring surgery), and sepsis, which might affect long-term neurodevelopmental and growth outcomes^2^. ORs for respiratory morbidities were adjusted for antenatal steroids therapy, surfactant use, gestational age, SGA, intraventricular hemorrhage (≥ grade 3), periventricular leukomalacia, necrotizing enterocolitis (≥ stage 2), retinopathy of prematurity (requiring surgery), and sepsis, which might affect long-term respiratory outcomes. The long-term outcomes were considered as binary dependent variables, and the BPD definition was considered as a categorical independent variable in each model. A GEE method was applied to find the association between the severity of BPD and long-term outcomes over time (follow-up 1 and 2). *P*-values for trend were analyzed to evaluate the trend between long-term outcomes and the severity of BPD. *P*-values < 0.05 were considered statistically significant. Statistical analyses were conducted using SAS version 9.4 (SAS Inc., Cary, NC, USA).

### Ethics statement

The institutional review board of each participating hospital reviewed and approved the KNN registry during admission and follow-up, and written informed consent was obtained from the infants’ parents at enrollment. ‘The Korean Neonatal Network ethics committee’ approved the present study. All the methods were performed in accordance with the approved protocol. The names of the institutional review board of the KNN participating hospitals were as follows: The institutional review board of Ajou University Hospital, Asan Medical Center, Busan ST Mary’s Hospital, CHA Bundang Medical Center, CHA University, CHA Gangnam Medical Center, CHA University, Cheil General Hospital & Women’s Healthcare Center, Chonbuk National University Hospital, Chonnam National University Hospital, Chosun University Hospital, Chung-Ang University Hospital, Chungbuk National University, Chungnam National University Hospital, Daegu Catholic University Medical Center, Dong-A University Hospital, Dongguk University Ilsan Hospital, Eulji General Hospital, Eulji University Hospital, Ewha Womans University Medical Center, Gachon University Gil Medical Center, Gangnam Severance Hospital, GangNeung Asan Hospital, Gyeongsang National University Hospital, Hanyang University Guri Hospital, Hanyang University Medical Center, Inha University Hospital, Inje University Busan Paik Hospital, Inje University Haeundae Paik Hospital, Inje University Ilsan Paik Hospital, Inje University Sanggye Paik Hospital, Jeju National University Hospital, Kangbuk Samsung Hospital, Kangdong Sacred Heart Hospital, Kangnam Sacred Heart Hospital, Kangwon National University Hospital, Keimyung University Dongsan Medical Center, Konkuk University Medical Center, Konyang University Hospital, Korea University Anam Hospital, Korea University Ansan Hospital, Korea University Guro Hospital, Kosin University Gospel Hospital, Kyung Hee University Hospital at Gangdong, Kyung Hee University Medical center, Kyungpook National University Chilgok Hospital, Kyungpook National University Hospital, National Health Insurance Service Ilsan Hospital, Pusan National University Children’s Hospital, Pusan National University Hospital, Samsung Changwon Medical Center, Samsung Medical Center, Seoul Metropolitan Government-Seoul National University Boramae Medical Center, Seoul National University Bundang Hospital, Seoul National University Hospital, Severance Hospital, Soonchunhyang University Hospital Cheonan, Soonchunhyang University Hospital Bucheon, Soonchunhyang University Hospital Seoul, Sungae Hospital, The Catholic University of Korea Bucheon ST. Mary’s Hospital, The Catholic University of Korea Seoul ST. Mary’s Hospital, The Catholic University of Korea ST. Vincent’s Hospital, The Catholic University of Korea Yeouido ST. Mary’s Hospital, The Catholic University of Korea Uijeongbu ST. Mary’s Hospital, Ulsan University Hospital, Wonju Severance Christian Hospital, Wonkwang University School of Medicine & Hospital, and Yeungnam University Hospital.

## Supplementary Information

Supplementary Information.

## Data Availability

The dataset analyzed in this study is not publicly available due to the policy of the Korea National Institute of Health. However, datasets are available from the corresponding author upon reasonable request.
