# Supplementary material for: Comparison of definitions of bronchopulmonary dysplasia to reflect the long-term outcomes of extremely preterm infants
Source: Sci Rep. 2022 Oct 27;12:18095. doi: 10.1038/s41598-022-22920-8 (PMC9613988; doi:10.1038/s41598-022-22920-8)
Supplement: Supplementary file 1 — Supplementary Information. [file 41598_2022_22920_MOESM1_ESM.pdf]

Supplementary Table 1. Characteristics of the patients who were followed-up at corrected age of 18–24 months and 3 years of age

	Total N=2380	Follow-up 1 N=1508	Lost-to-follow-up 1 N=666	*P* value	Follow-up 2 N=938	Lost-to-follow-up 2 N=1209	*P* value
Gestational age, weeks	25.8±1.2	25.8±1.2	25.8±1.2	0.677	25.7±1.2	25.8±1.2	0.012
23, n (%)	114/2380 (4.8)	77/1,508 (5.1)	29/666 (4.4)	0.942	55/938 (5.9)	50/1,209 (4.1)	0.112
24, n (%)	289/2380 (12.1)	175/1,508 (11.6)	80/666 (12.0)		121/938 (12.9)	133/1,209 (11.0)	
25, n (%)	478/2380 (20.1)	303/1,508 (20.1)	133/666 (20.0)		196/938 (20.9)	238/1,209 (19.7)	
26, n (%)	663/2380 (27.9)	422/1,508 (28.0)	183/666 (27.5)		255/938 (27.2)	343/1,209 (28.4)	
27, n (%)	836/2380 (35.1)	531/1,508 (35.2)	241/666 (36.2)		311/938 (33.2)	445/1,209 (36.8)	
Birth weight, g	882.0±194.2	878.9±193.1	898.9±191.4	0.060	860.9±190.8	900.7±192.7	<0.001
<500 g, n (%)	45/2380 (1.9)	34/1,508 (2.3)	5/666 (0.8)	0.062	27/938 (2.9)	12/1,209 (1.0)	<0.001
500–<750 g, n (%)	570/2380 (24.0)	355/1,508 (23.5)	151/666 (22.7)		239/938 (25.5)	271/1,209 (22.4)	
750–<1000 g, n (%)	1096/2380 (46.1)	707/1,508 (46.9)	309/666 (46.4)		441/938 (47.0)	555/1,209 (45.9)	
1000–<1500 g, n (%)	669/2380 (28.1)	412/1,508 (27.3)	201/666 (30.2)		231/938 (24.6)	371/1,209 (30.7)	
Male, n (%)	1274/2380 (53.5)	800/1,508 (53.1)	363/666 (54.5)	0.531	501/938 (53.4)	653/1,209 (54.0)	0.782
Small for gestational age, n (%)	147/2380 (6.2)	102/1,508 (6.8)	25/666 (3.8)	0.006	69/938 (7.4)	59/1209 (4.9)	0.016
Apgar score at 1 min, median (IQR)	4 (3-5)	4 (3-5)	4 (3-5)	0.847	4 (2-5)	4 (3-5)	0.032
Apgar score at 5 min, median (IQR)	7 (5-7)	7 (5-8)	7 (5-7)	0.184	7 (5-7)	7 (5-7)	0.615
Antenatal steroids therapy, n (%)	1985/2338 (84.9)	1,279/1,480 (86.4)	552/656 (84.2)	0.166	802/921 (87.1)	1009/1,189 (84.9)	0.147
Maternal chorioamnionitis	1036/2043 (50.7)	708/1,336 (53.0)	254/547 (46.4)	0.010	440/844 (52.1)	506/1,017 (49.8)	0.307
Maternal GDM	165/2380 (6.9)	115/1,508 (7.6)	41/666 (6.2)	0.221	65/938 (6.9)	84/1,209 (7.0)	0.987
Maternal PIH	211/2380 (8.9)	143/1,508 (9.5)	47/666 (7.1)	0.065	93/938 (9.9)	96/1,209 (7.9)	0.109
Maternal level of education				<0.001			<0.001
Elementary school	5/2380 (0.2)	2/1,508 (0.1)	2/666 (0.3)		1/938 (0.1)	3/1,209 (0.3)	
Middle school	24/2380 (1.0)	10/1,508 (0.7)	13/666 (2.0)		2/938 (0.2)	22/1,209 (1.8)	
High school	452/2380 (19.0)	266/1,508 (17.6)	152/666 (22.8)		165/938 (17.6)	255/1,209 (21.1)	
University, or more	1363/2380 (57.3)	942/1,508 (62.5)	325/666 (48.8)		632/938 (67.4)	622/1,209 (51.5)	
Unknown	536/2380 (22.5)	288/1,508 (19.1)	174/666 (26.1)		138/938 (14.7)	307/1,209 (25.4)	

Values are expressed as mean ± standard deviation or number (%). IQR, interquartile range; GDM, gestational

diabetes mellitus; PIH, pregnancy induced hypertension.
